# Molecular epidemiology and clinical features of Hepatitis C Virus (HCV) in epidemic areas of Interior Sindh, Pakistan

**DOI:** 10.12669/pjms.325.10429

**Published:** 2016

**Authors:** Shameem Bhatti, Sobia Manzoor

**Affiliations:** 1 Shameem Bhatti, Atta-ur-Rahman School of Applied Bio-Sciences, Department of Healthcare Biotechnology, National University of Sciences and Technology (NUST), Islamabad 44000, Pakistan; 2 Sobia Manzoor, Atta-ur-Rahman School of Applied Bio-Sciences, Department of Healthcare Biotechnology, National University of Sciences and Technology (NUST), Islamabad 44000, Pakistan

**Keywords:** Hepatitis C virus (HCV), Genotype, Immuno-chromatographic Tests (ICT), Intravenous (IV), Hepatitis B virus (HBV)

## Abstract

**Objective:**

Highly variable genome of HCV and high prevalence in many geographical areas made it necessary to conduct local population studies. This study has been conducted to show HCV parameters along with clinical features in the local population of interior Sindh, province of Pakistan.

**Methods:**

Present study was conducted in from August 2010 to November 2015 in the rural areas of Sindh, Pakistan. All the 31560 screened samples selected for the study were tested by second Generation Enzyme Linked Immunosorbent Assay (ELISA Biokit 480&96).

**Results:**

Total 31560 people were screened for HCV and out of these 13.67% (n= 4314) HCV infected patients. When 4314 samples of patients were examined; the anti-HCV was significantly higher in males 2814 (14.98%) than in females 1500 (11.74%) with P value = 0.06. The age of the patients ranged from 18 to 65 years. Out of 4314 HCV samples, 3020 (70%) were of Genotype 3a, 237(5.5%) of Genotype 2a, 108 (2.5%) of Genotype- 1a, 216 (5%) of Genotype 1b, 237 (5.5%) of Genotype 3b and 43 (1%) of Genotype 4. Additionally, 108 (2.5%) had co-infection and 345 (8%) samples showed no result –designated as untypable by the genotyping.

**Conclusion:**

This study showed that HCV is most frequently reported disease with genotype 3a being the most prevalent genotype.

## INTRODUCTION

Hepatitis C Virus (HCV) is a chronic disease of liver which ultimately leads to Hepatic Steatosis, Cirrhosis and Liver Cancer.^1^ According to worldwide study, more than two hundred million people are infected by HCV.^2^ HCV is single-stranded (ss) RNA enveloped virus, which is being placed in family *flaviviridae*.^3^ Six major genotypes of HCV has been identified, which diverge by approximately 30-34% at the nucleotide sequence level, and several subtypes throughout the world. Most prevalent genotype 3a is very common in Pakistan.^4^ About 6% of the general population,^5^ approximately 17 million in Pakistan are infected with HCV^6^ however, HCV prevalence rate varies from 3 to 7% in different regions of the country.^7^ Almost eighty HCV cases out of hundred progress to chronic infection. Major risk factors in spread of HCV involve reuse of syringes and major/minor surgery/dental procedures.^8^

## METHODS

The present study was conducted from year 2010-2015 at Atta-ur-Rehman School of Applied Biosciences (ASAB), in collaboration with Gambat Institute of Medical Science College, District Khairpur Sindh, Pakistan. All the 31560 screened samples selected for the study were tested by second Generation Enzyme Linked Immunosorbent Assay (ELISA Biokit 480&96). The data obtained from these tests showed that 5028 (15.93%) subjects were positive for anti-HCV antibodies while only 16560 (52.5%) were negative by anti-HCV ELISA test. The results of qualitative PCR indicated that 4314 (13.70%) subjects were positive as compared to anti-HCV ELISA test where 5028 (15.93%) samples were anti-HCV positive. Thus 4314 patients were included for genotype assay and other biochemical analysis. Complete blood count was done using an Advia 2120i Hematology. In all patients, the levels of Serum alanine aminotransferase (ALT) and serum glutamic oxaloacetic transaminase (SGOT), Bilirubin level were done by using Advia 1800 chemistry Analyzer.

### Statistical Analysis

The statistical analysis of data was carried out by SPSS version 17. All variables results are given in the form of rates (%) and counts. P-Value equal to or less than 0.100 (at 10% level of Significance).

## RESULTS

A total of 31560 people were screened for hepatitis C in this study and out of these 13.67% (n= 4314) HCV infected patients in different Talukas of Gambat City. All HCV infected patients belonged to various regions of Gambat District Khairpur Sindh namely Gambat I, Gambat II, Kaamaldero, Jaado, Belhaaro, Khemmtia, Khoraa, Reepri, and Razeedero.

After screening 31560 subjects, number of patients found were 4314 samples of patients were examined; the anti-HCV antibodies were significantly higher in males 2814 (14.98%) than in females 1500 (11.74%) with P value 0.06.

The age of the patients ranged from 18 to 65 years. The HCV infection prevalence was highest in those who were aged above 41 years (19.40%) (Table-I). The results indicate that males are more at risk of getting infected with HCV at younger ages i.e. 41-45 (19.40%) than females who develop HCV infection in their middle ages 46-50 (13.44%).

**Table-I:** Prevalence of positivity for antibodies to hepatitis C virus in the sample, according to age groups (N = 31560).

*Age (Year)*	*Total Tested (N)*	*Males Tested (N)*	*Found Positive (%)*	*Females Tested (N)*	*Found Positive (%)*	*P-Value*
18-20	2072	1202	190 (15.80)	870	98 (11.26)	0.02
21-25	2962	1757	299 (17.01)	1205	125 (10.37)	0.15
26-30	4564	2871	401 (13.96)	1693	176 (10.40)	0.22
31-35	5038	2598	306 (11.77)	2440	316 (12.95)	0.47
36-40	5947	4054	576 (14.20)	1893	238 (12.57)	0.17
41-45	4633	2453	476 (19.40)*	2180	243 (11.46)	0.3
46-50	4231	2580	418 (16.20)	1651	222 (13.44)**	0.3
51-65	2113	1267	148 (11.68)	846	82 (9.69)	0.2
	31560	18782		12778		

* The results indicate that males are more at risk of getting infected with HCV at age i.e. 41-45 (19.40%) than female.

** Female develop more HCV infection in their ages 46-50 (13.44%)

HCV transmission was observed mainly associated with use of injections/drugs (23.20%) and reuse of syringes (13.42%), whereas 11.93% of HCV infected patients have reported sexual contact with HCV positive person. Out of total subjects, (32.55%) patients did not have common symptoms associated with HCV infection. While approximately 40.09% of all patients symptomatic history of fatigue and fever followed by abdominal pain and nausea (18.40%), and 8.96% had abdominal pain and fever (Table-II).

**Table-II:** Baseline characteristics of patients infected with HCV.

*Variable*	*Total Samples N=31560*	*Anti-HCV Positive (N=4314)*	*Anti-HCV Prevalence rate %*	*P-Value^g^*
Age (Years)				
≥35	14636	1911	13.06	0.02
≤36	16924	2403	14.20	
Sex^a^				
Male	18782	2814	14.98	0.06
Female	12778	1500	11.74	
Year of Study				
July 2010 ~ June 2013	9500	1140	12.00	0.13
August 2013 ~ March 2015	22150	3174	14.33	
Marital Statuse				
Married	25074	3659	14.59	0.16
Un-Married	5368	589	10.97	
Divorced/Separated	248	21	8.47	
Widow/Widower	870	45	5.17	
Socioeconomic Status				
Lower Class	17663	3059	17.32	0.10
Middle Class	12822	1184	9.23	
Upper Class	1075	71	6.60	
Educational Level				
High School or Higher	5586	541	9.68	0.05

Study shows that family contacts of HCV infected patients are at an increased risk of getting HCV infection by interfamilial contact. Majority of patients enrolled in this study had a hemoglobin level > 7.5-15 g/dL, a red blood cell count >3.8 x 106 /ml, a white blood cell count > 2500 up to 10,000 cells/mm3, and platelet count >100,000 cells/mm^3^. Measurement of the ALT level was increased with mean value estimated to be 80.31± 30.13 U/liter. Likewise, SGOT with mean value 60.91± 23.34 IU/liter and alkaline phosphatase level was increased with means value 252.62 ± 135.181 U/liter.

Out of 4314 HCV samples, 3020 (70%) were of genotype 3a, 237(5.5%) of genotype 2a, 108 (2.5%) of genotype 1a, 216 (5%) of genotype 1b, 237 (5.5%) of genotype 3b, and 43 (1%) of genotype 4. Additionally, 108 (2.5%) had co-infection and 345 (8%) samples showed no result i.e. untypable by the genotyping (Table-III).

**Table-III:** Distribution of HCV genotypes.

	*Types*	*Number (%)*	*P value^b^*
	3^a^	3020(70%)	
HCV Genotypinga	4^a^	43 (1%)	
	Co infection	108 (2.5%)	<0.5
	Untypable	345 (8%)	
	1^a^	108 (2.5%)	
	1^b^	216 (5%)	
	2^a^	237 (5.5%)	
	3^b^	237 (5.5%)	

a Genotype Values are the number of 4314 patients (percentage of the each type in the geographic region).

b Significant difference was observed with P-value of <0.5

## DISCUSSION

In Pakistan, HCV prevalence data is partly available, which makes it necessary to perform more community based HCV sero-prevalence study to get complete picture of HCV prevalence.^9^ HCV has become epidemic and increasing rapidly. Study shows that most of the HCV infections are linked with the use of syringes. It is probably because in Pakistan unnecessary injections are given commonly and based on the population point of view that intra venous injected medicine are extra effective as compared to oral medicine.^10^ Another Study also showed that those individuals who use increased number of injections are more likely to be HCV infected and these injections may be the main route of spread of HCV infection.^11^ Study results are in line with the research of Tariq, Husain et al. 1999,^12^ that males are more infected with HCV than females. Females are more infected in age 46-50 (13.44%) and males were more positive in age 41-45 (19.40%). High HCV positivity was observed in the middle age group of our study. Therefore, current survey is completely in accord with results of analysis of Muhammad and Jan 2005.^13^ where they detected high frequency of HCV amongst aged 40-50 years old people.

Center for Disease Control and Prevention has reported that around 18% of the HCV positive cases exist in people exposed to infected sexual partner.^14^ In our study, it has been observed that 11.93% of individuals reported sexual contact with HCV positive person. However, the exact frequency of sexually transmitted HCV infection in Pakistan cannot be determined because of religious and cultural reasons. International surveys suggested that the HCV spread might be high in male transvestites and female prostitutes.^15^

The present study shows that the family interactions of HCV infected cases are more significantly getting HCV infection by interfamilial contact. According to different studies conducted in Rome, India, Italy and Taiwan occurrence of HCV infection amongst household contacts, excluding cases with previous intra venous exposure was 3.6%, 16%, 7.3% and 8% respectively.^16^-^18^ In Egypt, it has been reported that transmission of HCV can occur between families other than spouses and showed highest incidence rate in children. The prevalence of HCV among children and family members is possible by exposure to infections, blood and syringes.^19^

It has also been observed that hypertensive and cardiac patients were infected with HCV. It was difficult to find any association with HCV and other diseases such as diabetes mellitus, malaria, typhoid, polio, tuberculosis and arthritis. Out of total patients, 32% HCV infection was asymptomatic, whereas, 40% patients were complaining of fatigue and fever during the course of infection.

This study indicates that distribution of genotype from the interior Sindh regions of Pakistan, genotype 3 is the most predominant form. A comparable epidemiological data on genotype distribution has been revealed in other regions (Including Bangladesh, Northern and Southern India and Nepal). The frequency of genotype 3 has been described as greater than 45% by^20^ and 70% in our study. Mostly people are unconscious about their symptoms and unaware about HCV positive. This study shows a high prevalence of HCV and established a high carrier state of clinically silent HCV infection.

## References

[1] Seeff LB (1999). Dilemma of the natural history of hepatitis C. J Gastroen Hepatol.

[2] Imran M, Manzoor S, Ashraf J, Khalid M, Tariq M, Khaliq HM (2013). Role of viral and host factors in interferon based therapy of hepatitis C virus infection. Virol J.

[3] Burbelo PD, Dubovi EJ, Simmonds P, Medina JL, Henriquez JA, Mishra N (2012). Serology enabled discovery of genetically diverse hepaciviruses in a new host. J Virol.

[4] Idrees M, Riazuddin S (2008). Frequency distribution of hepatitis C virus genotypes in different geographical regions of Pakistan and their possible routes of transmission. BMC Infect Dis.

[5] Hamid S, Umar M, Alam A, Siddiqui A, Qureshi H, Butt J (2004). PSG consensus statement on management of hepatitis C virus infection--2003. J Pak Med Assoc.

[6] Idrees M, Riazuddin S (2008). Frequency distribution of hepatitis C virus genotypes in different geographical regions of Pakistan and their possible routes of transmission. Bmc Infect Dis.

[7] Zuberi SJ (1996). Seroepidemiology of HBV/HCV in Pakistan. Int Hepatol Comm.

[8] Idrees M, Lal A, Naseem M, Khalid M (2008). High prevalence of hepatitis C virus infection in the largest province of Pakistan. J Dig Dis.

[9] (2004). Transmission of hepatitis B virus in correctional facilities--Georgia, January 1999-June 2002. MMWR Morb Mortal Wkly Rep.

[10] Janjua NZ, Hutin YJ, Akhtar S, Ahmad K (2006). Population beliefs about the efficacy of injections in Pakistan’s Sindh province. Public Health.

[11] Jafri W, Jafri N, Yakoob J, Islam M, Tirmizi SF, Jafar T (2006). Hepatitis B and C:prevalence and risk factors associated with seropositivity among children in Karachi, Pakistan. BMC Infect Dis.

[12] Tariq WU, Hussain AB, Karamat KA, Ghani E, Hussain T, Hussain S (1999). Demographic aspects of hepatitis C in northern Pakistan. J Pak Med Assoc.

[13] Muhammad N, Jan MA (2005). Frequency of hepatitis “C” in Buner, NWFP. J Coll Physicians Surg Pak.

[14] Manavi M, Baghestanian M, Watkins-Riedel T, Battistutti W, Pischinger K, Schatten C (2002). Detection of hepatitis C virus (HCV) RNA in normal cervical smears of HCV-seropositive patients. Clin Infect Dis.

[15] Umar M, Hamama-tul-Bushra Ahmad M, Khurram M, Usman S, Arif M (2010). Hepatitis C in Pakistan:A Review of Available Data. Hepat Mon.

[16] Ho MS, Yang CS, Chen PJ, Mau YC (1994). Intrafamilial transmission of hepatitis C virus. J Clin Microbiol.

[17] Nakashima K, Ikematsu H, Hayashi J, Kishihara Y, Mitsutake A, Kashiwagi S (1995). Intrafamilial Transmission of Hepatitis-C Virus among the Population of an Endemic Area of Japan. JAMA.

[18] Sood A, Midha V, Sood N, Awasthi G (2002). Prevalence of anti-HCV antibodies among family contacts of hepatitis C virus-infected patients. Indian J Gastroenterol.

[19] Mohamed MK, Abdel-Hamid M, Mikhail NN, Abdel-Aziz F, Medhat A, Magder LS (2005). Intrafamilial transmission of hepatitis C in Egypt. Hepatology.

[20] Afridi S, Naeem M, Hussain A, Kakar N, Babar ME, Ahmad J (2009). Prevalence of hepatitis C virus (HCV) genotypes in Balochistan. Molecular Biology Reports.

